# Supplementary material for: A Likelihood Approach for Real-Time Calibration of Stochastic Compartmental Epidemic Models
Source: PLoS Comput Biol. 2017 Jan 17;13(1):e1005257. doi: 10.1371/journal.pcbi.1005257 (PMC5240920; doi:10.1371/journal.pcbi.1005257)
Supplement: S1 File — (TAR.GZ) [file pcbi.1005257.s014.tar.gz › HSPH_Online-SI-Revision/EnKF07/n10-severe/n10-severe_EnKF_table-8.pdf]

1	R0	{1.68952, 11.8402}	{2.32999, 5.97306}	{{1.47911, 2.42548}, {3., 10.0099}}	{44.4713, 49.5527}	inf	217.	333.823	{177.477, 716.919}	93.1315	0.260715	newly	155.	619.03	{86., 1500.}	334.806	0.428863	Reff	1.60166	0.627721	{0.239597, 1.48134}	62.4111	ar	6872.	18 460.8	{17 021., 23 218.}	168.638
2	R0	{1.71739, 9.62661}	{2.004, 4.65665}	{{0.163746, 0.868011}, {3., 6.01989}}	{64.2029, 58.9517}	inf	89.	382.567	{112.866, 581.601}	333.616	0.54781	newly	86.	413.26	{84., 809.}	387.326	0.564041	Reff	1.66844	0.276828	{0.024203, 0.461055}	83.408	ar	6874.	15 794.1	{14 656., 17 270.}	133.359
3	R0	{1.48936, 6.23975}	{2.33342, 4.86401}	{{1.74913, 0.931002}, {3., 13.1209}}	{59.6846, 62.2308}	inf	75.	2388.04	{504.648, 5478.06}	3085.32	1.32868	newly	102.	2874.47	{482., 7410.}	2719.4	1.22855	Reff	1.45406	0.608932	{0.197788, 1.34259}	59.2605	ar	5666.	15 180.8	{7640., 24 686.}	169.919
4	R0	{1.62394, 7.51135}	{2.16587, 9.09664}	{{0.49875, 5.81488}, {3., 14.5361}}	{70.0049, 49.1112}	inf	51.	449.989	{318.161, 618.579}	790.143	0.872245	newly	60.	320.04	{112., 589.}	443.5	0.680649	Reff	1.59877	0.364877	{0.0963192, 0.574544}	77.1776	ar	6417.	17 912.9	{16 910., 19 817.}	179.147
5	R0	{1.53632, 8.57652}	{2.12915, 8.91335}	{{0.488142, 5.80378}, {3., 14.5454}}	{74.0837, 45.3091}	inf	91.	465.589	{314.095, 654.034}	415.116	0.619569	newly	100.	359.04	{114., 644.}	264.06	0.421573	Reff	1.48962	0.385953	{0.0421448, 0.684699}	74.0905	ar	6260.	18 040.4	{16 938., 20 104.}	188.185
6	R0	{1.47166, 5.7434}	{2.34802, 5.40799}	{{1.4957, 2.39495}, {3., 10.0262}}	{59.5876, 64.4561}	inf	45.	319.112	{173.524, 560.999}	614.902	0.71441	newly	63.	664.57	{92., 2247.}	957.952	0.653745	Reff	1.45297	0.649153	{0.239121, 1.49555}	59.2385	ar	5650.	18 200.9	{16 775., 22 174.}	222.14
7	R0	{1.48179, 8.87369}	{2.11459, 8.992}	{{0.456188, 5.88169}, {3., 14.3886}}	{80.9124, 44.1287}	inf	250.	422.547	{33.5727, 310.57}	97.1143	0.306935	newly	229.	316.33	{12., 302.}	86.214	0.352815	Reff	1.37169	0.351426	{0.0455839, 0.591318}	74.3801	ar	5233.	17 639.	{16 826., 17 937.}	237.072
8	R0	{1.53669, 6.47807}	{2.17679, 4.30877}	{{0.988101, 2.67836}, {3., 5.1328}}	{63.0489, 37.4489}	inf	77.	1596.61	{913.187, 5229.27}	1974.67	1.17167	newly	107.	1643.82	{687., 5433.}	1438.88	1.02727	Reff	1.50611	0.320864	{0.0519496, 0.486121}	78.6958	ar	5934.	15 479.6	{6924., 26 100.}	165.796
9	R0	{1.57227, 6.20989}	{2.29192, 9.87086}	{{0.768067, 2.02303}, {3., 16.9591}}	{61.8306, 76.1629}	inf	9.	2602.85	{538.767, 5789.85}	28 824.6	2.28664	newly	13.	2775.77	{418., 8532.}	21 257.5	2.09102	Reff	1.56598	1.36808	{0.348848, 2.21928}	34.1706	ar	6136.	13 184.3	{8906., 17 313.}	115.347
10	R0	{1.54936, 6.50938}	{2.24391, 11.2572}	{{1.26763, 6.37596}, {3., 16.9544}}	{57.9927, 85.1887}	inf	30.	2619.96	{361.764, 5789.84}	8636.64	1.74305	newly	40.	2331.53	{327., 4185.}	5735.63	1.52344	Reff	1.53789	1.37158	{0.240597, 2.21924}	34.7858	ar	6275.	12 588.	{6244., 17 651.}	104.36
11	R0	{1.42179, 3.18601}	{2.17627, 6.17557}	{{0.859608, 2.15937}, {3., 10.0502}}	{75.199, 108.138}	inf	3.	2858.34	{807.849, 5378.24}	95 177.9	2.8558	newly	12.	3473.19	{1172., 6967.}	28 843.3	2.30635	Reff	1.40601	1.09599	{0.341961, 2.16712}	37.9643	ar	5322.	13 347.4	{8139., 19 568.}	151.364
12	R0	{1.48035, 6.46183}	{2.18969, 6.27371}	{{0.864726, 2.15979}, {3., 10.0701}}	{73.0758, 45.8682}	inf	26.	2878.17	{665.537, 5139.22}	10 971.8	1.8868	newly	35.	3455.77	{741., 6312.}	9775.97	1.80339	Reff	1.47147	1.06212	{0.351, 2.1056}	40.8825	ar	5793.	13 198.8	{7612., 19 752.}	130.542
13	R0	{1.75755, 8.53854}	{2.18834, 4.56532}	{{1.1887, 2.67802}, {3., 5.12638}}	{50.5353, 48.9905}	inf	33.	1617.86	{614.07, 5455.88}	4802.6	1.53858	newly	37.	1852.35	{668., 5377.}	4907.11	1.49411	Reff	1.74472	0.425988	{0.0436157, 0.880377}	77.6896	ar	6832.	16 042.8	{7641., 24 508.}	139.026
14	R0	{1.47355, 4.89451}	{2.57651, 7.61854}	{{2.13206, 4.13554}, {3., 14.6721}}	{77.2918, 64.3461}	inf	20.	2032.	{646.497, 3437.35}	10 060.5	1.84894	newly	45.	1948.25	{479., 4936.}	4230.96	1.45865	Reff	1.4625	0.883403	{0.343516, 2.0432}	52.2243	ar	5710.	14 892.8	{10 371., 21 386.}	161.729
15	R0	{1.62071, 5.98665}	{1.74501, 7.9398}	{{0.382033, 2.00079}, {3., 17.4943}}	{56.1555, 91.1934}	inf	11.	710.942	{120.303, 1431.56}	6363.11	1.61407	newly	20.	2130.1	{89., 5517.}	10 552.4	1.61778	Reff	1.61763	1.53759	{0.261855, 2.87977}	53.8344	ar	6635.	6741.74	{1562., 10 551.}	50.1872
16	R0	{1.67268, 7.7256}	{2.48346, 7.15427}	{{2.12885, 3.94792}, {3., 14.6951}}	{50.7177, 43.6505}	inf	103.	2039.37	{340.734, 4546.09}	1885.5	1.15554	newly	121.	2241.71	{288., 5865.}	1758.02	1.07722	Reff	1.63555	0.93612	{0.342731, 2.06274}	52.4366	ar	6947.	14 561.8	{8393., 21 344.}	111.28
17	R0	{1.67805, 11.1417}	{2.56047, 7.24887}	{{2.13365, 4.0617}, {3., 14.7052}}	{54.8248, 43.9092}	inf	109.	2142.52	{641.186, 4256.85}	1868.7	1.1613	newly	87.	2273.	{578., 6659.}	2514.44	1.22848	Reff	1.646	0.924406	{0.342891, 2.14587}	54.3958	ar	6625.	14 892.7	{8409., 22 639.}	125.113
18	R0	{1.66743, 9.99046}	{2.12541, 4.22166}	{{0.989925, 2.67893}, {3., 5.14642}}	{54.4082, 57.7431}	inf	118.	1518.76	{914.459, 3911.82}	1188.11	0.992297	newly	114.	1705.52	{701., 5361.}	1397.51	1.02213	Reff	1.61574	0.370116	{0.0520941, 0.794877}	78.077	ar	6899.	15 781.9	{7295., 23 379.}	133.996
19	R0	{1.70787, 9.85478}	{2.07725, 8.98199}	{{0.482772, 5.79754}, {3., 14.5368}}	{65.9095, 43.0422}	inf	143.	559.839	{53.1462, 1203.1}	310.402	0.536939	newly	120.	432.95	{20., 862.}	281.475	0.492676	Reff	1.63409	0.382513	{0.04099, 0.580749}	76.5983	ar	6867.	18 286.9	{16 970., 22 397.}	166.302
20	R0	{1.53809, 10.5848}	{2.1193, 8.84061}	{{0.493694, 5.80554}, {3., 14.5756}}	{75.6533, 42.2419}	inf	127.	567.563	{306.744, 1002.74}	358.283	0.535808	newly	110.	459.78	{112., 1100.}	330.764	0.465809	Reff	1.48364	0.385386	{0.0412892, 0.631445}	74.0243	ar	6108.	18 355.1	{16 977., 23 405.}	200.51
21	R0	{1.59925, 6.74479}	{2.10412, 10.8632}	{{0.879652, 4.2696}, {3., 16.959}}	{49.6202, 74.5471}	inf	35.	2485.09	{556.367, 4526.65}	7002.05	1.68534	newly	45.	2166.07	{427., 4072.}	4717.49	1.47489	Reff	1.58662	1.25617	{0.239377, 2.13751}	33.771	ar	6411.	12 425.8	{6975., 17 246.}	97.6458
22	R0	{1.38779, 4.40977}	{2.48418, 7.13688}	{{2.12821, 3.88488}, {3., 14.7071}}	{80.2225, 69.4244}	inf	43.	2049.23	{489.108, 4480.}	4667.8	1.519	newly	75.	2263.53	{328., 5819.}	2920.95	1.27952	Reff	1.36101	0.934208	{0.343051, 2.04288}	50.0897	ar	5154.	14 604.9	{9051., 21 267.}	184.108
23	R0	{1.53657, 7.50352}	{2.39816, 5.01044}	{{1.67624, 1.12892}, {3., 13.1511}}	{61.0961, 60.7369}	inf	100.	2523.22	{502.05, 5478.59}	2423.22	1.23747	newly	116.	2944.46	{497., 7909.}	2438.33	1.22526	Reff	1.50353	0.663865	{0.212927, 1.3461}	56.8389	ar	5928.	15 628.8	{7119., 25 681.}	166.021
24	R0	{1.71131, 6.24526}	{2.59472, 7.58986}	{{2.13153, 4.17257}, {3., 14.6891}}	{53.3063, 45.2902}	inf	12.	2125.87	{645.334, 3492.05}	17 616.7	2.10783	newly	18.	2064.62	{605., 4939.}	11 371.4	1.89189	Reff	1.70104	0.903221	{0.32505, 2.1095}	54.9227	ar	6924.	15 053.2	{10 366., 22 105.}	119.082
25	R0	{1.4412, 3.07729}	{2.25886, 9.27759}	{{0.608864, 2.10802}, {3., 13.2074}}	{78.4258, 213.856}	inf	5.	1891.83	{684.065, 4126.28}	37 736.7	2.43885	newly	20.	2339.73	{426., 7129.}	11 598.7	1.82812	Reff	1.43789	1.55293	{0.248615, 2.72801}	47.8276	ar	5810.	12 043.6	{5193., 19 500.}	113.209
26	R0	{1.4159, 4.93858}	{2.25625, 10.2756}	{{0.886055, 2.02062}, {3., 16.9489}}	{71.9807, 120.841}	inf	18.	2410.59	{161.131, 4466.63}	13 297.2	1.93008	newly	30.	2576.46	{84., 6341.}	8496.73	1.68291	Reff	1.40925	1.38469	{0.301199, 2.219}	37.6389	ar	5255.	12 937.3	{6519., 17 832.}	147.483
27	R0	{1.57465, 7.05414}	{2.21356, 9.13247}	{{0.590641, 2.10802}, {3., 14.9172}}	{62.8154, 63.7728}	inf	15.	1980.43	{684.065, 4356.62}	13 102.9	2.00905	newly	22.	2592.45	{465., 7973.}	11 683.9	1.84866	Reff	1.57134	1.51598	{0.256012, 2.6786}	41.9224	ar	6359.	11 752.4	{5396., 18 422.}	90.4079
28	R0	{1.41422, 4.62402}	{2.19395, 9.62269}	{{1.37079, 2.0203}, {3., 16.9377}}	{66.0986, 122.12}	inf	6.	2387.73	{538.296, 4203.04}	39 698.2	2.41498	newly	14.	2549.87	{414., 6321.}	18 118.4	2.02208	Reff	1.41153	1.33828	{0.388224, 2.21896}	37.3226	ar	5485.	12 785.2	{7316., 17 380.}	134.109
29	R0	{1.41901, 3.63017}	{1.71244, 7.62459}	{{0.331799, 2.00079}, {3., 16.1394}}	{68.1084, 149.083}	inf	3.	706.841	{119.985, 1365.47}	23 461.4	2.18655	newly	4.	2261.04	{165., 7191.}	56 426.	2.32983	Reff	1.4183	1.5348	{0.296968, 2.88194}	60.9904	ar	5979.	6574.86	{1550., 10 547.}	59.3082
30	R0	{1.60719, 4.72984}	{1.73198, 7.60804}	{{0.381408, 2.00079}, {3., 16.1415}}	{57.669, 110.809}	inf	1.	700.123	{119.774, 1362.34}	69 912.3	2.65786	newly	2.	2281.5	{186., 7107.}	113 975.	2.64372	Reff	1.60687	1.55511	{0.312447, 2.8823}	52.6423	ar	6479.	6656.95	{1501., 10 525.}	52.6842
31	R0	{1.38175, 6.26468}	{2.31878, 5.5187}	{{0.869264, 2.15989}, {3., 7.78445}}	{83.6243, 38.2822}	inf	64.	3128.1	{1172.01, 5602.72}	4787.65	1.59221	newly	96.	4038.3	{1205., 8680.}	4106.56	1.49199	Reff	1.35577	1.10051	{0.404229, 1.72126}	33.3939	ar	5397.	13 510.6	{6814., 19 829.}	152.817
32	R0	{1.4947, 8.8847}	{2.12565, 9.38012}	{{0.464583, 5.8192}, {3., 14.4686}}	{77.7961, 45.7858}	inf	208.	454.351	{57.789, 418.258}	143.421	0.337883	newly	186.	350.85	{21., 411.}	131.952	0.383714	Reff	1.38021	0.39879	{0.0750015, 0.666157}	71.1065	ar	5407.	17 953.7	{16 910., 20 020.}	232.045
33	R0	{1.55891, 8.28978}	{2.26284, 9.68627}	{{0.479369, 5.88495}, {3., 14.5186}}	{77.1504, 49.1381}	inf	149.	508.052	{66.0769, 648.589}	258.838	0.498891	newly	142.	379.84	{35., 549.}	191.169	0.398605	Reff	1.49203	0.407553	{0.0936375, 0.675684}	72.899	ar	6239.	18 152.4	{16 926., 20 577.}	190.95
34	R0	{1.60764, 10.4729}	{2.1325, 9.44206}	{{0.468383, 5.97105}, {3., 14.4741}}	{71.0703, 40.7176}	inf	256.	442.849	{35.6541, 308.626}	104.486	0.327792	newly	188.	336.14	{12., 357.}	126.457	0.413053	Reff	1.49125	0.378064	{0.058392, 0.66239}	74.6478	ar	6495.	17 826.9	{16 854., 18 801.}	174.471
35	R0	{1.48527, 5.74485}	{2.27128, 6.09364}	{{0.866151, 2.15955}, {3., 10.0679}}	{75.8618, 46.8677}	inf	25.	2785.25	{769.895, 5089.17}	11 043.4	1.90229	newly	43.	3404.98	{1028., 5968.}	7822.74	1.7384	Reff	1.47606	1.09067	{0.351254, 2.1444}	40.0633	ar	5811.	13 317.	{8261., 19 745.}	132.297
36	R0	{1.61457, 9.93364}	{2.02667, 4.41013}	{{0.163972, 0.891767}, {3., 5.95901}}	{70.4087, 60.7119}	inf	112.	455.292	{129.366, 712.244}	311.268	0.496621	newly	110.	497.4	{105., 932.}	358.764	0.501722	Reff	1.56904	0.271653	{0.0245753, 0.459622}	82.6866	ar	6338.	16 651.1	{15 499., 18 135.}	163.382
37	R0	{1.47831, 7.08139}	{2.57826, 7.00032}	{{2.13003, 4.03756}, {3., 10.8189}}	{75.6719, 42.0082}	inf	63.	2090.56	{644.646, 4254.05}	3218.59	1.38164	newly	82.	2251.33	{581., 5864.}	2645.52	1.25907	Reff	1.45954	0.920054	{0.389908, 2.14636}	52.4463	ar	5530.	15 076.2	{8388., 22 371.}	172.625
38	R0	{1.73445, 9.8858}	{2.57921, 6.98748}	{{2.13212, 3.98029}, {3., 10.8306}}	{50.3708, 40.4749}	inf	67.	2087.42	{650.296, 4258.97}	3015.63	1.35437	newly	71.	2250.42	{605., 5847.}	3069.61	1.32366	Reff	1.70548	0.919077	{0.389761, 2.14374}	54.4255	ar	6842.	14 973.1	{8305., 22 312.}	120.095
39	R0	{1.66621, 11.4824}	{2.47543, 5.71342}	{{1.2842, 4.20081}, {3., 7.79614}}	{55.0765, 50.242}	inf	179.	258.857	{15.8172, 263.856}	114.636	0.404033	newly	134.	263.81	{13., 457.}	147.276	0.409765	Reff	1.58806	0.505222	{0.178684, 1.08185}	71.156	ar	6473.	17 800.5	{16 542., 19 562.}	174.997
40	R0	{1.60694, 9.95741}	{2.50666, 5.92317}	{{1.18256, 4.23197}, {3., 7.88304}}	{62.2299, 40.5149}	inf	141.	191.771	{64.1483, 271.97}	73.5392	0.261744	newly	132.	209.79	{38., 478.}	95.9621	0.30856	Reff	1.54957	0.613186	{0.172518, 1.77277}	67.2753	ar	6625.	18 243.6	{16 578., 23 672.}	175.375
41	R0	{1.55128, 4.71911}	{1.70812, 7.78795}	{{0.280057, 2.00079}, {3., 16.1372}}	{61.2069, 114.921}	inf	6.	689.806	{120.195, 1362.76}	11 396.8	1.8785	newly	17.	2138.48	{170., 6075.}	12 482.1	1.70671	Reff	1.54988	1.53976	{0.273342, 2.9046}	56.3043	ar	6141.	6594.	{1526., 10 555.}	58.0212
42	R0	{1.60063, 6.81118}	{2.4559, 6.56574}	{{1.49344, 2.49939}, {3., 10.1187}}	{57.1223, 53.9774}	inf	35.	376.592	{173.925, 459.854}	981.295	0.800618	newly	65.	553.33	{85., 1864.}	757.185	0.582574	Reff	1.58799	0.552241	{0.0283801, 1.48011}	68.4923	ar	6528.	18 138.2	{16 783., 21 578.}	177.853
43	R0	{1.56986, 5.44652}	{2.20917, 9.14718}	{{0.590832, 2.10806}, {3., 14.9189}}	{63.2556, 96.8163}	inf	8.	1877.2	{683.931, 3721.05}	23 365.	2.25676	newly	23.	2497.18	{462., 7984.}	10 757.4	1.80923	Reff	1.56751	1.52044	{0.250372, 2.72635}	41.9504	ar	6312.	11 791.2	{5351., 18 437.}	92.3216
44	R0	{1.47523, 8.07785}	{1.96459, 4.75792}	{{0.164843, 0.892621}, {3., 5.96102}}	{82.7599, 51.7334}	inf	101.	378.822	{104.113, 594.529}	282.259	0.499042	newly	100.	405.76	{86., 914.}	316.72	0.514746	Reff	1.43968	0.271599	{0.0243738, 0.460268}	81.1347	ar	5692.	16 055.5	{15 238., 17 353.}	183.111
45	R0	{1.6417, 7.69429}	{2.57936, 7.30125}	{{2.13469, 4.00828}, {3., 14.6539}}	{58.6507, 42.5419}	inf	45.	2078.18	{636.338, 4256.52}	4520.84	1.50777	newly	60.	2157.46	{457., 5177.}	3498.5	1.37034	Reff	1.62545	0.94122	{0.344414, 2.19749}	53.4539	ar	6735.	15 077.4	{8602., 22 115.}	124.612
46	R0	{1.38865, 5.83473}	{2.36897, 5.1935}	{{1.78284, 1.09838}, {3., 13.1792}}	{73.5423, 64.1285}	inf	64.	2312.06	{501.111, 5478.23}	3513.89	1.39654	newly	101.	2742.18	{483., 7355.}	2616.46	1.22843	Reff	1.35977	0.639831	{0.172808, 1.34407}	55.0985	ar	5233.	15 113.4	{7203., 24 667.}	190.622
47	R0	{1.45289, 6.87903}	{2.48953, 7.47271}	{{2.12879, 4.04198}, {3., 14.7034}}	{74.466, 46.0889}	inf	78.	2131.47	{643.458, 3544.55}	2634.72	1.304	newly	104.	2229.35	{612., 5206.}	2045.41	1.16067	Reff	1.42441	0.971436	{0.306323, 2.1987}	51.8989	ar	5379.	14 690.9	{8659., 22 155.}	173.116
48	R0	{1.69416, 9.4831}	{2.05934, 4.56895}	{{0.164964, 0.883102}, {3., 5.86442}}	{62.0057, 57.8817}	inf	47.	427.371	{128.186, 609.354}	810.482	0.806802	newly	41.	468.56	{97., 833.}	1045.61	0.860485	Reff	1.67264	0.278117	{0.0234476, 0.460279}	83.3726	ar	6925.	16 233.7	{15 167., 17 511.}	134.822
49	R0	{1.47843, 7.16286}	{2.38248, 5.26603}	{{1.6662, 1.06828}, {3., 13.2084}}	{66.1228, 60.53}	inf	80.	2518.24	{497.564, 5486.8}	3047.8	1.3278	newly	81.	2819.55	{479., 7982.}	3380.93	1.34858	Reff	1.44664	0.641738	{0.23525, 1.34434}	56.4958	ar	5449.	15 643.6	{7728., 25 608.}	189.451
50	R0	{1.68843, 5.72427}	{1.70918, 8.03122}	{{0.332067, 2.00079}, {3., 16.2715}}	{55.2547, 94.4725}	inf	14.	686.602	{120.616, 1366.1}	4804.6	1.51362	newly	26.	2037.19	{157., 5405.}	7739.04	1.507	Reff	1.68522	1.53035	{0.273449, 2.90406}	53.3382	ar	6800.	6598.65	{1555., 10 593.}	50.5225
